# Introducing the MCHF/OVRP/SDMP: Multicapacitated/Heterogeneous Fleet/Open Vehicle Routing Problems with Split Deliveries and Multiproducts

**DOI:** 10.1155/2014/515402

**Published:** 2014-06-16

**Authors:** Duygu Yilmaz Eroglu, Burcu Caglar Gencosman, Fatih Cavdur, H. Cenk Ozmutlu

**Affiliations:** Department of Industrial Engineering, Uludag University, Gorukle, 16059 Bursa, Turkey

## Abstract

In this paper, we analyze a real-world OVRP problem for a production company. Considering real-world constrains, we classify our problem as multicapacitated/heterogeneous fleet/open vehicle routing problem with split deliveries and multiproduct (MCHF/OVRP/SDMP) which is a novel classification of an OVRP. We have developed a mixed integer programming (MIP) model for the problem and generated test problems in different size (10–90 customers) considering real-world parameters. Although MIP is able to find optimal solutions of small size (10 customers) problems, when the number of customers increases, the problem gets harder to solve, and thus MIP could not find optimal solutions for problems that contain more than 10 customers. Moreover, MIP fails to find any feasible solution of large-scale problems (50–90 customers) within time limits (7200 seconds). Therefore, we have developed a genetic algorithm (GA) based solution approach for large-scale problems. The experimental results show that the GA based approach reaches successful solutions with 9.66% gap in 392.8 s on average instead of 7200 s for the problems that contain 10–50 customers. For large-scale problems (50–90 customers), GA reaches feasible solutions of problems within time limits. In conclusion, for the real-world applications, GA is preferable rather than MIP to reach feasible solutions in short time periods.

## 1. Introduction

Open vehicle routing problems (OVRPs) have gained much attention recently since they represent a problem type that needs to be solved by many production companies. In most industries, companies choose to use a hired vehicle fleet for distributing their goods. In this way, they do not have to endure the extra cost for returning vehicles since they use the resources of a third-party logistics (3PL) provider such as trucks or TIRs [1]. As consequences of such benefits, however, the companies have to accept some restrictions defined by the 3PL providers. For example, the 3PL provider can determine some particular routes considering the experiences of the drivers or the highway conditions. In addition, the 3PL provider can restrict the number of customers visited by a particular vehicle. Such specifications require some changes in the classical OVRP structure to make them more applicable for real-world problems.

In this paper, we consider a real-world vehicle routing problem for a production company. The company produces two different types of products (multiple products—MP) with different volume and weight properties. The first product is a lightweight but large product, such as styrofoam, and the second product has opposite volume-weight characteristics, heavyweight but small product, such as tar. Since the company uses a fleet of vehicles of a 3PL provider, the vehicles do not need to return to the depot, and thus, the underlying problem becomes an open vehicle routing problem (OVRP). The 3PL provider has a heterogeneous fleet of vehicles, such as trucks and TIRs (heterogeneous fleet—HF), with different volume and weight capacities (multiple capacitated—MC). In addition, on any route, the vehicles can serve two customers at most, and the demand of a customer can be supplied by different vehicles (split delivery—SD). Based on these problem specifications, we classify our problem as a multicapacitated/heterogeneous fleet/open vehicle routing problem with split deliveries and multiproducts (MCHF/OVRP/SDMP), which, to the best of our knowledge, has not yet been considered in previous studies.

Since the OVRP consists of Hamiltonian paths, we should find the best Hamiltonian path for each set of customers assigned to a vehicle for the optimal solution [2]. Therefore, it can be concluded that the OVRP has an NP-hard structure because of the NP-hard Hamiltonian path subproblems. Many researchers have developed various heuristic approaches to solve the OVRP. Brandão [2] developed a tabu search algorithm for the OVRP to generate good solutions in short time periods where the researcher obtained better results using a random tabu tenure instead of a fixed one [2]. Zachariadis and Kiranoudis [3] developed a novel approach to produce initial solutions for OVRPs. The researchers tested their metaheuristic on some well-known OVRP instances where improvements on several best-known solutions from previous studies were presented [3]. Eksioglu et al. [4] presented an extensive literature review for the vehicle routing problem (VRP), classified the VRP studies according to their specifications, and built the taxonomy of the VRP literature. According to the study, the VRP literature was categorized into five main groups that contain 106 different subcategories. They discovered that the number of the VRP studies including load specific and heterogeneous vehicles has been three times less than the others. We note that there have been a few VRP studies that contain heterogeneous vehicles in the literature. In this study, we consider an OVRP with heterogeneous fleet where we evaluate the loading process using the volume and weight coefficients of the products which has not yet been studied in detail so far.

Capacitated open vehicle routing problems (COVRPs) have also been studied by various researchers. Among these, Letchford et al. [5] formulated the first exact algorithm, and Simbolon [6] developed a direct search algorithm for the COVRP. In our study, we additionally consider the volume and weight capacities (multicapacitated—MC) of a heterogeneous fleet (HF) of vehicles. Just like the multicapacitated problems, the heterogeneous fleet of vehicles is also common in real-world transportation problems since a smaller vehicle with sufficient capacity would always be preferable to a larger one which, beyond decreasing the transportation cost, is also a basic principle of green logistics. Some studies considered different variations of these problems, such as Gendreau et al. [7] and Taillard [8] who developed a tabu search algorithm and a column generation method to solve the heterogeneous fleet vehicle routing problem (HVRP), respectively. The study of Tavakkoli-Moghaddam et al. [9] additionally considered the concept of split service in the capacitated vehicle routing problem (CVRP) where they showed that splitting demand implies a higher capacity utilization. In split delivery, customers might be visited by more than one vehicle similar to some real-life scenarios especially in the existence of a 3PL provider. Many researchers have studied the vehicle routing problems with split delivery properties. The study of Archetti and Speranza [10] presented a survey of split delivery vehicle routing problems (SDVRP) that includes the description of the SDVRP, its properties, exact algorithms and heuristics, and its variants and applications. None of the above studies, however, considered the open vehicle routing problem (OVRP) with a multiple capacitated heterogeneous fleet and multiple products with split deliveries together as in our study.

This paper is organized as follows. In Section 2, a mixed-integer programming (MIP) model is formulated. In Section 3 the proposed hybrid genetic-local search is described. Computational results and conclusions are presented in Sections 4 and 5, respectively.

## 2. Mixed-Integer Programming Model

In this section, we present a mixed-integer programming model for the MCHF/OVRP/SDMP. A real-world problem of production company with its real-life assumptions was considered for the model. As mentioned before, the company uses a heterogeneous fleet of vehicles of a 3PL provider for distribution of goods, and the 3PL provider allows the vehicles to serve two customers at most. The 3PL provider determines the transportation cost considering the target customer. In addition, if a vehicle supplies the demand of an intermediate customer along with the target customer, the 3PL provider charges a stopping cost which depends on the vehicle type. We therefore calculate the total cost of the distribution process considering the transportation and the stopping cost of the vehicles together, which is aimed to be minimized in the MIP model. In our experiments, we consider two different types of products, however we define the type of materials with “*m*” index, and we allow MIP to model the problems with multiple types of materials (more than two) simultaneously. The notation of the MIP model can be found in Table 1. Consider

(1) $$\begin{array}{c} \min\;z=\overset{}{\underset{(i,j,k)\in \text{Se}{\text{t}}_{1}\;|\;i=j}{\sum }}C_{jk}*y_{ijk}+\underset{\left(i,j,k\right)\in \text{Se}{\text{t}}_{1}\;|\;i\neq j}{\sum }F_{ik}*y_{ijk}, \end{array}$$

(2) $$\begin{array}{c} \underset{(i,j,k)\in \text{Se}{\text{t}}_{1}\;|\;i=j}{\sum }y_{ijk}\leq 1,\;\forall k\in K, \end{array}$$

(3) $$\begin{array}{c} \underset{k\in K}{\sum }y_{ijk}\leq 1,\;\forall i,j\in I\;|\;i=j, \end{array}$$

(4) $$\begin{array}{c} \underset{i\in I\;|\;(i,j,k)\in \text{Se}{\text{t}}_{1},i\neq j}{\sum }y_{ijk}\leq y_{jjk},\;\forall j\in I,\;k\in K, \end{array}$$

(5) $$\begin{array}{c} x_{ijkm}\leq L*y_{ijk},\;\forall i,j\in I,\;k\in K,\;m\in M, \end{array}$$

(6) $$\begin{array}{c} \underset{j\in I}{\sum }\;\underset{k\in K}{\sum }x_{ijkm}=D_{im},\;\forall i\in I,\;m\in M, \end{array}$$

(7) $$\begin{array}{c} \underset{i\in I}{\sum }\;\underset{j\in J}{\sum }\;\underset{m\in M}{\sum }W_{m}*x_{ijkm}\leq G_{k},\;\forall k\in K, \end{array}$$

(8) $$\begin{array}{c} \underset{i\in I}{\sum }\;\underset{j\in J}{\sum }\;\underset{m\in M}{\sum }V_{m}*x_{ijkm}\leq P_{k},\;\forall k\in K, \end{array}$$

(9) $$\begin{array}{c} x_{ijkm}\geq 0\;\text{and}\;\text{integer}, \end{array}$$

(10) $$\begin{array}{c} y_{ijk}\in \left\{0,1\right\},\;\forall i\in I,\;j\in I,\;k\in K. \end{array}$$

The objective function (1) aims for the minimization of total cost which includes the transportation and the stopping cost of vehicles. To reduce the size of the solution space, we determine the maximum number of vehicles for the problem, and use this knowledge as a parameter for the index of vehicles. Constraint (2) ensures that each vehicle has only one target customer at most. Constraint (3) shows that each customer is decided as a target by only one vehicle at most. Constraint (4) guarantees that a vehicle can serve two customers at most; one, if applies, is the intermediate customer and the other is the target customer. The relationship between decision variables has been set by constraint (5). Constraint (6) ensures that the demands of customers are met by the vehicles. Constraints (7) and (8) represent the capacities of the vehicles in terms of weight and volume, respectively. Finally, (9) and (10) present the definition space of the decision variables.

Since the definition of the problem is unique, no benchmark for this type of problem is available. We therefore generate random samples for evaluating the performance of the MIP model which are detailed in Section 4.1. Although we can find the solutions with smaller gaps of small-size problems (10–50 customers) using MIP, it is not able to find any feasible solution of large-size real-world-like problems (60–90 customers) within time limits (7200 s). To handle large-size problems, we develop a genetic algorithm with local search which is detailed in Section 3.

## 3. Hybrid Genetic-Local Search Algorithm

A genetic algorithm (GA) is a search technique based on the biological process of evolution theory that mimics natural selection. One of the important issues in GA is the genetic representation (string of symbols) of each solution in a population. The string is referred to as* chromosome* and the symbols as* genes* [11]. After generation of the initial population and determination of the fitness function values for each chromosome, GA manipulates the selection process by operations such as reproduction, crossover, and mutation [11]. The introduction of GA dates back to 1970s [12]. As the searching technique of genetic algorithms (GAs) [13] became popular in the mid-1980s, many researchers started to apply the approach to different types of problems. To date, GA has been applied to many different types of problems including vehicle routing problems. In this paper, we also have developed a hybrid genetic-local search algorithm for the MCHF/OVRP/SDMP problem which is detailed in the next section. The proposed local search algorithms, implemented in C#, are performed during the fitness function calculation process. In order to eliminate the infeasible chromosome structure, we were mainly inspired by our previous algorithms related to scheduling problem in the literature [14, 15]. The novel characteristic of the studied problem is the capacity constraint. The algorithm is not only applied to logistic area but also included capacities of vehicles and orders. The developed new approach related to capacity issue is integrated into the algorithm successfully and is explained in the next section.

### 3.1. Genetic Algorithm for Order Splitting Property

#### 3.1.1. Encoding Scheme

**Figure 1:**
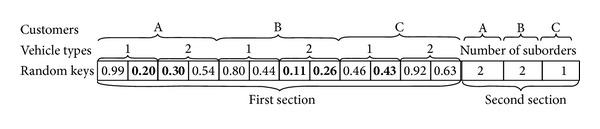
Representation of a chromosome in the GA.

In the proposed GA, a chromosome is mainly designed by a string of random numbers that are uniformly generated between 0 and 1. These random keys show the transportation route for each vehicle. In addition, each chromosome also carries the number of suborders for each customer. It would be necessary to note that this chromosome structure is used in our previous study [14].  Figure 1 illustrates a sample chromosome. In the first section of the chromosome, the string contains *n* (number of customers) segment. Each segment is further divided into *m* (number of vehicle types) part, and then, each part for vehicle types is divided into genes. The number of genes for vehicles is two in this problem, which shows the maximum number of suborders. Note that the chromosome structure is convenient to increase the maximum suborder quantities. To decide the exact number of suborders for each customer, it is necessary to check the second section of the chromosome. In the second section of the chromosome, the string contains *n* (number of customers) segment which shows the number of suborders for each customer. These numbers might be 1 or 2 for this problem, and they are generated randomly to reflect the variable suborder environment. For the chromosome structure in Figure 1, we have 2 vehicle types and 3 customers. For *Customer*_*A*_, since the number of suborders equals 2 as in the second section's first segment of the chromosome, the smallest two random numbers will be selected from the first section of the chromosome among all the numbers generated for *Customer*_*A*_. The selected values (0.2 and 0.3 for *Customer*_*A*_) are in bold in Figure 1. It means that the first order of *Customer*_*A*_ will be transported by *VehicleType*_1_ and the second order of *Customer*_*A*_ will be transported by *VehicleType*_2_. Similarly, the number of suborders is 2 for *Customer*_*B*_ and they will be transported by *VehicleType*_2_. The number of suborders is 1 for *Customer*_*C*_ and it will be transported by *VehicleType*_1_.

#### 3.1.2. Fitness Function

**Figure 2:**
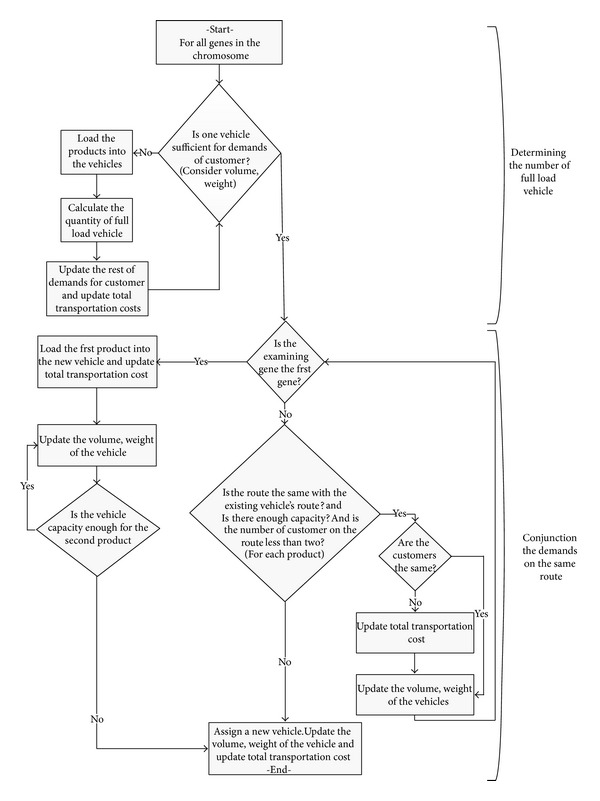
Determining the number of vehicles and their loads.

**Figure 3:**
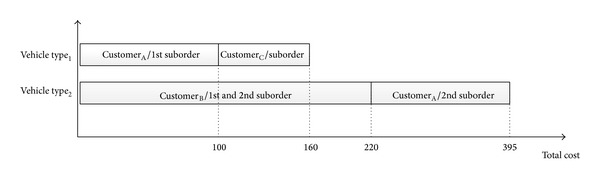
Resulting loading schema for chromosome in Figure 1.

In the proposed algorithm, the chromosome structure defines the routes for each vehicle type. But we also need to integrate capacity conditions into the algorithm.  Figure 2 shows the outline of integration process. We consider the volume and weight constraints of vehicles as well as the demands of each customer. After obtaining the routes for the customers—which will be explained in detail shortly after as in Figure 3—we need to iterate the integration process in two phases as shown in Figure 2. In the first phase, if the demands of customers are greater than a full load vehicle, then the number of fully loaded vehicles and costs are calculated. In the second phase of the algorithm, the remaining demands of customers are trying to be consolidated to maximize vehicles' utilizations. After assigning the first demand to the vehicle, if the capacity of the existing vehicle is enough, route is the same, and the number of customers on the route is less than two, then the next demand can be loaded into the vehicle. This loading demand might be the suborder of the same product, the demand of the second product of the same customer, or the demand of the next customer on the route. Otherwise, the new vehicle is required. To explain how the algorithm obtains the routes for the customers and calculate total costs, the example in Figure 1 is considered. According to the chromosome shown in Figure 1, the number of suborders for *Customer*_*A*_, *Customer*_*B*_, and *Customer*_*C*_ is determined as two, two, and one, respectively. *VehicleType*_1_ will supply the 1st suborder of *Customer*_*A*_ and the suborder of *Customer*_*C*_. *VehicleType*_2_ will supply the 1st and the 2nd suborders of *Customer*_*B*_ and the 2nd suborder of *Customer*_*A*_. The sequence of customers on *VehicleType*_1_ will be determined by the random key numbers (0.20 and 0.43). Increasing arrangement of these random numbers will designate *VehicleType*_1_'s customer sequence. Similarly, the sequence of customers on *VehicleType*_2_ will be determined by the random key numbers (0.11, 0.26, and 0.30). Figure 3 shows the vehicle types and the customers for the examining problem. According to Figure 2, we need to calculate the full load vehicles for each customer's suborders, considering two types of products. Then for the remaining loads, we need to combine the vehicles on the same route. For the considered example, three customers and two vehicle types, we will ignore the vehicles which are fully loaded in order to simplify the explanation.

As mentioned earlier, two types of costs are considered in the problem: stopping costs and transportation costs for each type of vehicle. For the considered example, each vehicle type's route and customers' loads (information about splitting or not splitting) are shown in Figure 3. Assume that Table 2 shows the stopping costs for each type of vehicle, and Tables 3 and 4 present the transportation costs of *VehicleType*_1_ and *VehicleType*_2_, respectively. Transportation costs from the depot to the corresponding customers are shown in the first rows of Tables 3 and 4. The total cost is calculated by utilizing the transportation and stopping costs. Considering the first vehicle of *VehicleType*_1_, the transportation cost from depot to *Customer*_*A*_ is 60, and the stopping cost of *VehicleType*_1_ is 40, so the partial total cost is 60 + 40 = 100, and then the same vehicle transports from *Customer*_*A*_ to *Customer*_*B*_ and the transportation cost is 20. Similarly the stopping cost of *VehicleType*_1_ is 40. It means that the total cost of *VehicleType*_1_ is 100 + 20 + 40 = 160. The total cost of *VehicleType*_2_ is greater than the total cost of *VehicleType*_1_. Our aim is to minimize the maximum total cost. As seen in Figure 3, the fitness value of chromosome in Figure 1 is 395. The structure of chromosome will be modified with the aim of finding the routes with minimum cost utilizing local search. The details of the GA-based local search algorithm are explained in Section 3.2. After the algorithm runs, we obtain the total cost for the chromosome. The population size determines the number of different chromosomes and fitness function values.

#### 3.1.3. Genetic Operators

After generating the initial population, the operations of selection, crossover, and mutation are iteratively used to search for the best solution.

**Figure 4:**
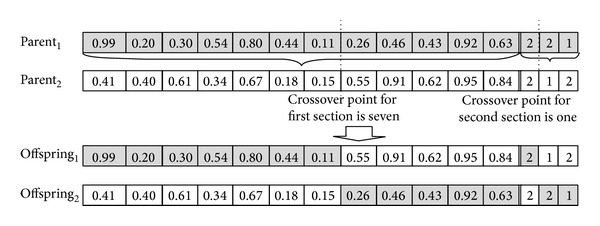
Crossover operation: Two parents mate in order to produce two offspring.

**Figure 5:**
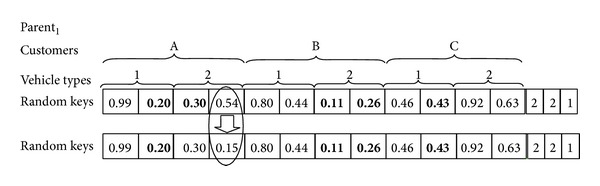
Mutation operation.

- Selection: chromosomes are selected into the mating pool based on the random selection method [16]. In this method, parents are randomly chosen from the population.
- Crossover: the crossover operator, with crossover rate *P*_*c*_, is a method for sharing information between chromosomes. We use single point crossover approach in our algorithm which randomly chooses the crossing point and exchanges the genes between two parents to create the offspring. We use separate crossover operations in the first and second phases.  Figure 4 illustrates the crossover operation.
- Mutation: the mutation operator is used to prevent the algorithm converging to a local optimum. In our algorithm, a mutation operation is performed as follows. Mutation operation is applied to the randomly selected chromosomes of the population according to the mutation rate (*P*_*m*_). The value of randomly selected gene of the selected chromosome is replaced with a new random number. By applying the operation to all selected chromosomes, we can obtain new chromosomes with new routes and fitness function values. The fitness value might be better or worse or remain same after applying the operator. An example for our problem is shown in Figure 5.

To enhance the performance of the genetic algorithm, we use a local search algorithm in the literature [14], which is detailed in the next subsection.

### 3.2. Local Search

Integrating one of the local search techniques within GA will commonly generate more competitive results. For instance, the integrating dominance properties method that was originally developed by Chang and Chen [17] was afterward applied successfully to machine scheduling problem [15]. In the other paper of researchers, which includes job splitting property in scheduling problem [14], an advance approach is adopted. And in this paper, finally the algorithm adopted more complex aforementioned logistics problem which contains capacity constraints. By searching all possible alternatives in a chromosome, we aim to reduce the total cost. After this process, the routes will be determined.

We consider all alternative situations for the local search as summarized in the Appendix. The proposed intervehicle type (interchange) and intravehicle type (exchange) customer changes are explained in Section 3.2.1 and the calibration of random numbers are explained in Section 3.2.2. The notation used in the pseudocode and some explanations on the local search operation are as follows.

**Figure 6:**
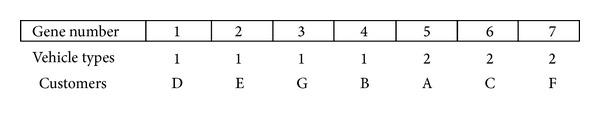
Sample gene structure for an example that contains seven customers and two vehicle types.

- *g*: the selected genes from the chromosome are ordered by vehicle type and then by customer sequence. The value of *g* shows the total number of these genes. Each individual gene contains vehicle type and customer features.  Figure 6 explains a sample gene structure of an example with seven customers and two vehicle types. The structure also indicates the sequence of customers for each type of vehicle.
- *i* and *j*: they are the gene numbers which are compared to decide whether the customers encoded in these genes should be exchanged or not.
- *Gene*(*i*)_*VehicleType*_: it indicates the type of vehicle encoded in the *i*th gene. According to the structure shown in Figure 6, the type of vehicle for the 1st gene (*Gene*(1)_*VehicleType*_) is 1.
- *Gene*(*i*)_*Customer*_: it indicates the customer encoded in the *i*th gene. According to the structure shown in Figure 6, the customer for the 1st gene (*Gene*(1)_*Customer*_) is Customer D.
- *TC*_*VehType*(*i*),*Customer*(*j*,*k*)_: it is the transportation cost between *Gene*(*j*)_*Customer*_ and *Gene*(*k*)_*Customer*_ for *Gene*(*i*)_*VehicleType*_.
- *UC*_*VehType*(*i*),*Customer*(*j*)_: it is the stopping cost of *Gene*(*i*)_*VehicleType*_ for *Gene*(*j*)_*Customer*_.
- *TT*_*Gene*(*i*)*VehicleType*_: it is the total transportation cost for *Gene*(*i*)_*VehicleType*_.

#### 3.2.1. Interchange and Exchange of Customers

- Interchange of customers: there are two cases to be considered within the intervehicle type interchange: adjacent customers interchange and nonadjacent customers interchange.
  - Adjacent interchange: in the intervehicle type, adjacent customers interchange section of the pseudocode, steps are as follows.
    - If *Gene*(*i* − 1)_*Customer*_ precedes *Gene*(*i*)_*Customer*_, then “a” represents the total difference of transportation costs between after interchange and before interchange situations.
    - If *Gene*(*i*)_*Customer*_ is the first customer on the vehicle, then “b” represents the total difference of transportation costs between after interchange and before interchange situations.
    - Either “a” or “b” must be equal to 0.
    - If *Gene*(*j*)_*Customer*_ precedes *Gene*(*j* + 1)_*Customer*_, then “c” represents the differences of transportation costs between after interchange and before interchange situations.
    - If the sum of “a,” “b,” and “c” is smaller than 0, it means that a lower total transportation cost is obtained. In this case, *Gene*(*i*)_*Customer*_ and *Gene*(*j*)_*Customer*_ are interchanged.
    - Calibrate the random key numbers. In Section 3.2.2, item (i) gives the description of the intervehicle type interchange.
  - Nonadjacent interchange: the steps for determining the exchange of *Ge*n*e*(*i*)_*Customer*_ and *Gene*(*j*)_*Customer*_ on the route are similar to adjacent interchange with one exception. During the calculation of “a” and “b,” the additional difference of transportation costs should be considered. The remaining steps are the same with the adjacent interchange situation.
- Intravehicle type exchanging: in the intravehicle type exchanging section of the pseudocode, steps are as follows.
  1. Calculate the total transportation cost of *Gene*(*i*)_*VehicleType*_ and *Gene*(*j*)_*VehicleType*_ before exchange. “D1" represents the maximum transportation cost of them.
  2. If *Gene*(*i* − 1)_*Customer*_ precedes *Gene*(*i*)_*Customer*_ on *Gene*(*i*)_*VehicleType*_, then “a1" represents the difference of transportation costs between after interchange and before interchange situations. If *Gene*(*i*)_*Customer*_ is the first customer on *Gene*(*i*)_*VehicleType*_, the second “a1" in the pseudocode represents the total difference of transportation costs between after interchange and before interchange situations.
  3. If *Gene*(*j* − 1)_*Customer*_ precedes *Gene*(*j*)_*Customer*_ on *Gene*(*j*)_*VehicleType*_, then “b1” represents the difference of transportation costs between after interchange and before interchange situations. If *Gene*(*j*)_*Customer*_ is the first customer on *Gene*(*j*)_*VehicleType*_, the second “b1” in the pseudocode represents the total difference of transportation costs between after interchange and before interchange situations.
  4. If *Gene*(*j*)_*Customer*_ precedes *Gene*(*j* + 1)_*Customer*_ on *Gene*(*j*)_*VehicleType*_, then “b2” represents the difference of transportation costs between after interchange and before interchange situations.
  5. Calculate the total transportation cost of *Gene*(*i*)_*VehicleType*_ and *Gene*(*j*)_*VehicleType*_ after a possible exchange. “D2” represents the maximum of these two values.
  6. If “D2” is smaller than “D1”, it means a lower total transportation cost is obtained. In this case, *Gene*(*i*)_*Customer*_ and *Gene*(*j*)_*Customer*_ are interchanged.
  7. Calibrate the random key numbers. In Section 3.2.2, item (ii) gives the description of intravehicle type exchange.

#### 3.2.2. Calibration of the Random Numbers on the Chromosome

**Figure 7:**
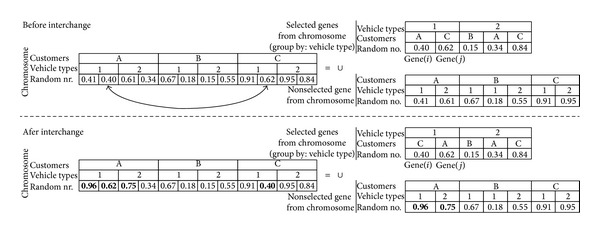
An example for the calibration of the random numbers on the chromosome for intervehicle type interchanges.

**Figure 8:**
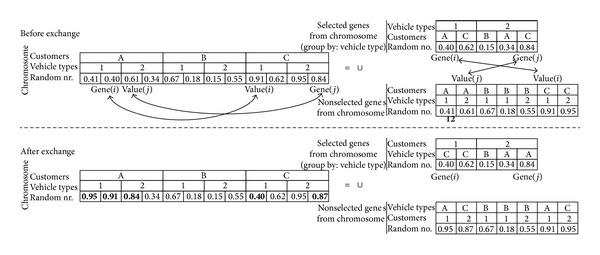
An example for the calibration of the random numbers on the chromosome for intravehicle type exchanges.

If it is decided to change *Gene*(*i*)_*Customer*_ and *Gene*(*j*)_*Customer*_, we need to calibrate the chromosome to represent the new situation by the following configurations. During the integration process, the positions of the random numbers are considered. In Figure 4, parent 2 is taken into account to explain the calibration phase. The orders of *Customer*_*A*_ and *Customer*_*C*_ are split into 2 suborders (no split for the orders of *Customer*_*B*_) for the considered chromosome. Due to simplicity, the sections of the number of suborders are not shown on the chromosomes of Figures 7 and 8.

- Intervehicle type interchange: Figure 7 shows the before and after interchange of the customers on the same vehicle type. The genes of a chromosome can be labeled as “selected genes from the chromosome” and “nonselected genes from the chromosome.” The realized interchanges are shown in bold on the after interchange section. The steps of interchanging *Gene*(*i*)_*Customer*_ and *Gene*(*j*)_*Customer*_ for the example are as follows.
  - Random numbers of the customers to be exchanged are 0.40 for *Gene*(*i*)_*Customer*_ → *Customer*_*A*_ and 0.62 for *Gene*(*j*)_*Customer*_ → *Customer*_*C*_ to be changed to 0.62 for *Gene*(*j*)_*Customer*_ → *Customer*_*A*_, and 0.40 for *Gene*(*i*)_*Customer*_ → *Customer*_*C*_.
  - After the interchange, 0.40 is smaller than 0.62, and hence there is no need to change other random numbers for *Customer*_*C*_. Because, the current situation guarantees *Customer*_*C*_ to select *VehicleType*_1_.
  - After the interchange, 0.62 is bigger than 0.40, and hence other random numbers for *Customer*_*A*_, which are smaller than 0.62 (as 0.41) in the “nonselected genes from chromosome,” must be changed. A new random number (bigger than 0.62) will be randomly generated (as 0.96) and changed to 0.41. Another random number, smaller than 0.62, is 0.61 in the “nonselected genes from chromosome” for *Customer*_*A*_. Therefore, a new random number (bigger than 0.62) will be randomly generated (as 0.75) and changed to 0.61.
- Intravehicle type exchange: Figure 8 shows the before and after exchange of the customers on two different vehicle types. The realized exchanges are shown in bold on the after exchange section of Figure 8. The steps of exchanging *Gene*(*i*)_*Customer*_ and *Gene*(*j*)_*Customer*_ for the example of Figure 8 are as follows. Value(*i*) is a maximum random number value for *Gene*(*j*)_*Customer*_ on *Gene*(*i*)_*VehicleType*_. Similarly, Value(*j*) is a maximum random number value for *Gene*(*i*)_*Customer*_ on *Gene*(*j*)_*VehicleType*_.
  - The random numbers for *Gene*(*i*) and Value(*i*) are exchanged in the chromosome. Also, the random numbers for *Gene*(*j*) and Value(*j*) are exchanged in the chromosome.
  - Between “selected genes from chromosome” and “nonselected genes from chromosome,” customers for *Gene*(*i*) and Value(*i*) are exchanged. And similarly customers for *Gene*(*j*) and Value(*j*) are exchanged.
  - After the exchange, it must be checked if there is a random number in the “nonselected genes from chromosome” for *Gene*(*j*)_*Customer*_ → *Customer*_*A*_ that is smaller than the random number of *Gene*(*j*) (equal to 0.84) or other selected value of *Customer*_*A*_ (equal to 0.34). For example, 0.41 is smaller than 0.84. Therefore, another random number (bigger than 0.84) will be randomly generated (as 0.95) and changed to 0.41.
  - It must also be checked if there is a random number in the “nonselected genes from chromosome” for *Gene*(*i*)_*Customer*_ → *Customer*_*C*_ that is smaller than the random number of *Gene*(*i*) (equal to 0.40) or other selected value of *Customer*_*C*_ (equal to 0.62). For example, 0.61 is smaller than 0.62. Thus, another random number (bigger than 0.62) will be randomly generated (as 0.87) and changed to 0.61.

As mentioned before, we consider a real-world MCHF/OVRP/SDMP problem. We first model the real system with mixed-integer programming, and then we develop a hybrid genetic-local search algorithm. We determine the required parameters and their values for the algorithms. We compare these algorithms with randomly generated problems using real-world parameters and detail the computational results in the next section.

## 4. Computational Results

### 4.1. Dataset

We consider the distribution network of a production company in Turkey. The company produces two types of products and uses a fleet of vehicles of a 3PL provider for distribution of goods to customers. In order to evaluate the performance of MIP and GA, we use the real-world data which are supplied from the 3PL provider. We gather the information of transportation costs, vehicle types and specialties, and properties of products from the 3PL provider, which are presented in Tables 5, 6, and 7, respectively.

We investigate the real-world demands and determine the distribution of them for two products as seen in Table 8. We represent the connections between customers with a 0-1 “Route (*R*_*ij*_)” matrix as in MIP model. The *R*_*ij*_ matrix is defined by the 3PL provider considering various constrains such as highway conditions or experiences of drivers. To generate different problems, we determine the *R*_*ij*_ matrix randomly for each problem. We develop *n* × *n* matrix for *n* customers and define the cells by *U*(0,1) distribution. If the *R*_*ab*_ value of customer *a* and customer *b* is 1, it means that there is a connection between the customer *a* and customer *b*. We also correct the diagonal of the matrix which should be 1. The generated examples can be found in [18].

### 4.2. Experimental Results

We generate different sizes of problems with different number of customers (varies from 10 to 90) which can be found in [18]. All experiments are run on a personal computer with an Intel Core i7-3612QM processor running at 2.10 GHz. We use C# for the GA implementation and CPLEX 12.4 for the MIP model. Before comparing MIP and GA, we first analyze the performance of MIP with different time limits. We limit MIP with 1800, 3600, and 7200 seconds and represent the results as MIP_18, MIP_36, and MIP_72, respectively. In the real-world application, the company has more than 50 customers. Although we prepare samples up to 90 customers, we could not use all of them since MIP_7200 could not find any feasible solution within time limits for the problem with 60 customers. Therefore, we restrict Table 9 with the samples up to 50 customers. We increase the number of samples for small-size problem, but the gap% gets bigger after 30 customers and problems get harder to solve; thus we generate only one problem for more than 30 customers. The gap% for the MIP models is calculated by the branch and bound algorithm in CPLEX 12.4 as in (11), where the best solution is represented by *z*′ and the best integer solution is represented by z*. The experimental results are detailed in Table 9,

(11) $$\begin{array}{c} \text{Gap}\%=\frac{\left|z^{\prime }-z^{*}\right|}{e^{-10}+\left|z^{*}\right|}. \end{array}$$

The first part of Table 9 gives the information about samples, the second part presents the objective function values found within different time limits, the third part shows the required time for solutions, and the fourth part indicates the gap% values which are calculated by CPLEX 12.4. The last part shows the improvements in percentage between time limits; the first column (18–36) presents the improvement in total cost if we run the MIP model within 3600 seconds instead of 1800 seconds, the second column (18–72) presents the improvement in total cost if we run the MIP model within 7200 seconds instead of 1800 seconds, and the last column (36–72) presents the improvement in total cost if we run the MIP model within 7200 seconds instead of 3600 seconds.

Table 9 indicates that the MIP model solves the small-size problems (10 customers) optimally within minutes. When the number of customers increases to 15, MIP generates similar results with all time limits, but it could not prove the optimality of solutions within time limits. Considering the problems that contain 20–50 customers, the difference between time limits becomes clear, and MIP_7200 performs better than the other compared options. In addition, MIP_7200 reaches solutions with smaller gap which are close to optimality as seen in the gap% part. Although the solutions look similar with different time limits, the improvement part clarifies the contribution of 7200 time limits to model results. If we use 3600 seconds instead of 1800 seconds, we improve the results with an average of 1.15%. However, if we use 7200 seconds, we improve the results with an average of 2.38%. Moreover, the real-world applications usually contain more than 50 customers, and the MIP_7200 model would provide better feasible results for larger examples; thus we limit MIP within 7200 seconds for next comparisons.

We compare the MIP model with the GA by using same randomly generated samples in previous experiment [18]. We limit MIP within 7200 seconds and determine the generation limits for GA as 500 generations. We detail the comparison between MIP and GA in Table 10. The first column shows the number of customers in each problem, the second column indicates the number of problems, the third part demonstrates the objective/fitness function of the MIP and GA, and the fourth part indicates the solution time of algorithms. We calculate the gap% for GA similar with the branch and bound algorithm by using the MIP result as the best solution in (12). The last two parts demonstrate the number of vehicles used by the algorithms. We define an upper bound for the vehicles in order to limit the vehicle index in the MIP model; however, the GA does not need such an upper bound:

(12) $$\begin{array}{c} \text{Gap}\%=\frac{Method_{Solution}-Best_{Solution}}{Best_{Solution}}*100. \end{array}$$

Experimental results show that MIP is able to solve the problems with 10 customers to optimality. However, for the problems that contain 20–50 customers, MIP could not reach the optimal solutions within time limits (2 hours). Moreover, MIP could not provide any feasible solution in 2 hours for large-size problems that contain 60–90 customers. On the other hand, GA is able to reach similar results with a 9.66% gap. In addition, the GA provides feasible solutions for larger problems which have similar size with real-world applications. The last three results of GA's elapsed time are higher than 7200 seconds, which are shown in* Italic*. It is possible to decrease the elapsed time via decreasing the number of generations. For example, if the same algorithm would run with 250 generations instead of 500 generations for the problem of 70 customers, the elapsed time would be 3817.24 seconds and total cost would be 85.660. Therefore, we can change the parameters of GA in order to catch the flexibility of real-world applications, and we can arrange them to reach feasible solutions within desirable times. In conclusion, the number of customers increases, the problem becomes harder to solve for both MIP and GA. However for the real-world applications, GA is preferable rather than MIP to reach feasible solutions in short time periods, by arranging the algorithm's parameters.

## 5. Conclusions and Directions for Future Research

In this paper, we introduce a vehicle routing problem, the MCHF/OVRP/SDMP, which has not yet been investigated in detail so far. With the aim of minimizing the total distribution cost, we have formulated a MIP model for the problem and developed a solution approach based on a genetic-local search algorithm requiring much less computational time than the MIP. Since the problem type discussed in this paper is not exactly the same as those in the literature, we randomly generated different problems with different routes and demands. We used these problems to evaluate the MIP model and the GA. The experimental results showed that although MIP was able to find better solutions than GA for small-size problems, it was not possible to obtain any feasible solutions of large-size problems within time limits. On the other hand, GA was able to reach feasible solutions of large-size problems in shorter time periods. However, the gap% between GA and MIP is greater than expected, and therefore, we continue our research to improve GA and to investigate new solution methods for this unique vehicle routing problem. Generating hybrid algorithms through the use of different metaheuristics might be a worthwhile avenue of research.

## Figures and Tables

**Algorithm 1:**
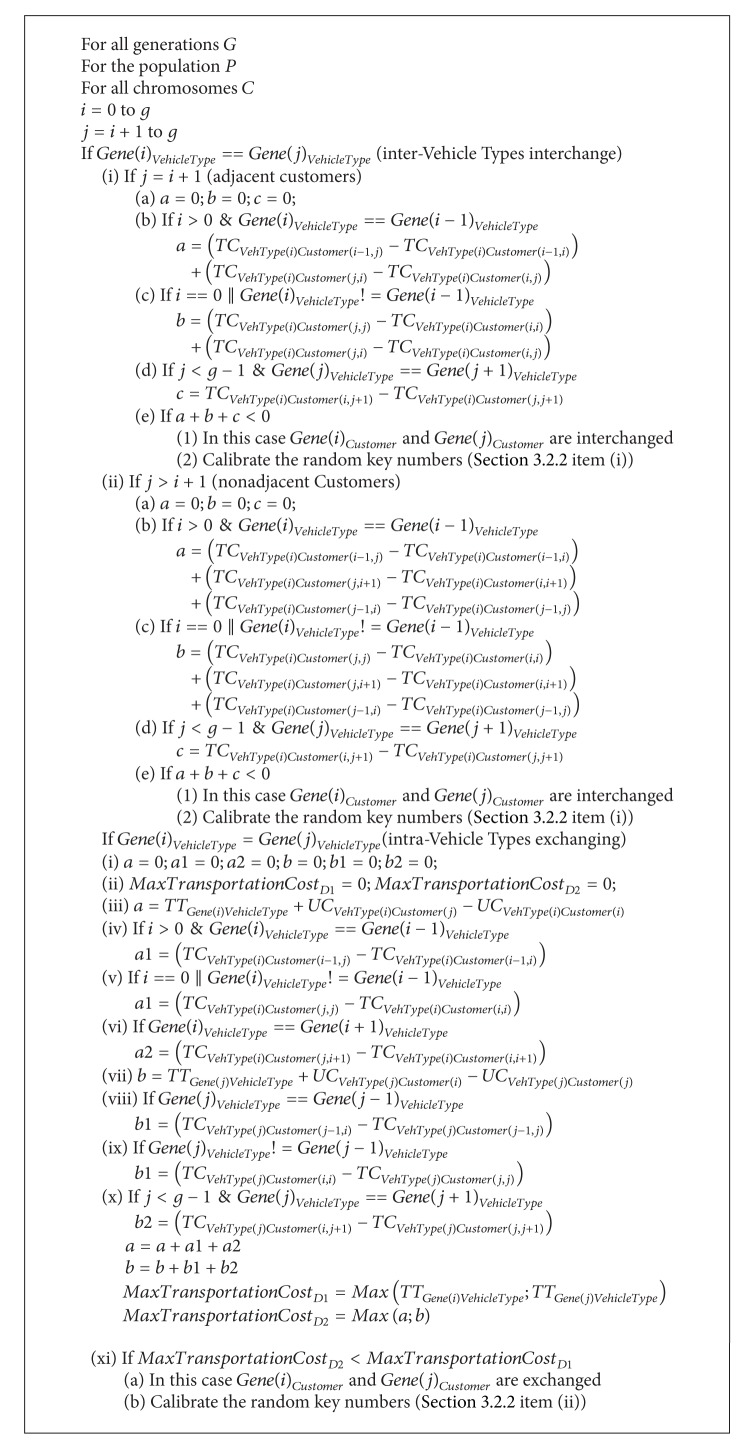
Local search.

**Table 1:** Sets, indices, parameters, and variables for MIP.

Indices (for sets see below):	
*i*, *j*	Index of customers, *i*, *j* ∈ *I*.
*k*	Index of vehicles, *k* ∈ *K*.
*m*	Index of product types, *m* ∈ *M*.
Parameters:	
*R*_*ij*_	0-1 matrix for connections of customers. *R*_*ij*_ is 1, if there is a connection between customer *i* and customer *j*.
*D*_*jm*_	The demand of product *m* for customer *j*.
*C*_*jk*_	Transportation cost for vehicle *k* while traveling to target city *j*.
*F*_*ik*_	Stopping cost for vehicle *k* on customer *i*.
*W*_*m*_	Weight coefficient for product *m*.
*V*_*m*_	Volume coefficient for product *m*.
*G*_*k*_	Weight capacity for vehicle *k*.
*P*_*k*_	Volume capacity for vehicle *k*.
*L*	Large number.
Sets:	
*I*	The set of customers, *I* = {1,2,…, *I*_max⁡_}.
*K*	The set of vehicles, *K* = {1,2,…, *K*_max⁡_}.
*M*	The set of product types, *M* = {1,2,…, *M*_max⁡_}.
Set_1_	{(*i*, *j*, *k*)∣*i* ∈ *I*, *j* ∈ *I*, *k* ∈ *K*, *R*_*ij*_ = 1}.
Decision variables:	
*x*_*ijkm*_	The quantity of product *m* that is left on customer *i*, while vehicle *k* is travelling to target customer *j*.
*y*_*ijk*_	$\{\begin{array}{cc} 1, & \text{if vehicle }k\text{ stops on customer }i\text{ while traveling to target customer }j;\\ 0, & \text{otherwise}. \end{array}$

**Table 2:** Stopping costs for each vehicle type.

*F*_*ik*_	*Cu* *st* *om* *er*_*A*_	*Cu* *st* *om* *er*_*B*_	*Cu* *st* *om* *er*_*C*_
*Ve* *hi* *cl* *eT* *yp* *e*_1_	40	40	40
*Ve* *hi* *cl* *eT* *yp* *e*_2_	80	80	80

**Table 3:** Transportation costs for *VehicleType*_1_.

*C*_*ij*1_	*Cu* *st* *om* *er*_*A*_	*Cu* *st* *om* *er*_*B*_	*Cu* *st* *om* *er*_*C*_
*Depot *	60	70	90
*Cu* *st* *om* *er*_*A*_	—	50	20
*Cu* *st* *om* *er*_*B*_	50	—	40
*Cu* *st* *om* *er*_*C*_	20	40	—

**Table 4:** Transportation costs for *VehicleType*_2_.

*C*_*ij*1_	*Cu* *st* *om* *er*_*A*_	*Cu* *st* *om* *er*_*B*_	*Cu* *st* *om* *er*_*C*_
*Depot *	120	140	160
*Cu* *st* *om* *er*_*A*_	—	95	50
*Cu* *st* *om* *er*_*B*_	95	—	80
*Cu* *st* *om* *er*_*C*_	50	80	—

**Table 5:** Stopping and transportation costs for the customers.

Vehicle type	Truck	TIR
*Stopping cost *	40$	80$
*Transportation cost *	1000$	1500$

**Table 6:** Volume and weight coefficient for each type of vehicle.

Capacity	Weight (kg)	Volume (m^3^)
*Tr* *uc* *ks*	15,500	45
*TI* *Rs*	25,000	84

**Table 7:** Volume and weight coefficient for each product.

Coefficient	Weight (kg)	Volume (m^3^)
*Pr* *od* *uc* *t*_1_	10.75	0.28
*Pr* *od* *uc* *t*_2_	1131	1.67

**Table 8:** Uniform distribution of demands of customers.

Product type	Distribution
*Pr* *od* *uc* *t*_1_	*U* (5; 250)
*Pr* *od* *uc* *t*_2_	*U* (2; 16)

**Table 9:** Comparison of MIP models with different time limits.

Customers	Problem	Total cost			Elapsed time			Gap%			Improvement%		
		MIP_18	MIP_36	MIP_72	MIP_18	MIP_36	MIP_72	MIP_18	MIP_36	MIP_72	18–36	18–72	36–72
10	1	9,400	9,400	9,400	340	340	340	0.00	0.00	0.00	0	0	0
	2	11,360	11,360	11,360	82	82	82	0.00	0.00	0.00	0	0	0
	3	10,700	10,700	10,700	131	131	131	0.00	0.00	0.00	0	0	0
	4	8,900	8,900	8,900	107	107	107	0.00	0.00	0.00	0	0	0
	5	11,820	11,820	11,820	100	100	100	0.00	0.00	0.00	0	0	0

15	1	18,180	18,140	18,140	1800	3600	7200	37.14	28.54	26.79	0.22	0.22	0
	2	14,140	14,100	14,100	1800	3600	7200	29.15	20.81	19.56	0.28	0.28	0
	3	13,600	13,600	13,600	1800	3600	7200	29.19	21.34	19.98	0	0	0
	4	16,180	16,100	16,100	1800	3600	7200	31.00	24.84	23.54	0.49	0.49	0
	5	15,020	15,020	15,020	1800	3600	7200	31.03	23.50	22.32	0	0	0

20	1	19,840	19,760	19760	1800	3600	7200	34.78	6.40	6.37	0.40	0.40	0
	2	22,540	21,960	21,880	1800	3600	7200	42.35	5.19	4.77	2.57	2.93	0.36
	3	20,420	19,380	19,380	1800	3600	7200	35.58	6.50	6.47	5.09	5.09	0
	4	21,220	20,800	20,800	1800	3600	7200	36.51	6.92	6.88	1.98	1.98	0
	5	19,380	18,260	18,260	1800	3600	7200	33.43	5.75	5.75	5.78	5.78	0

30	1	29,780	29,740	29,740	1800	3600	7200	23.65	22.81	21.96	0.13	0.13	0

40	1	42,920	41,300	41,300	1800	3600	7200	30.05	30.05	27.27	3.77	3.77	0

50	1	68,200	68,200	53,380	1800	3600	7200	51.98	51.96	38.62	0	21.73	21.73

							Avg.	24.77	8.47	3.02	1.15	2.38	1.23

**Table 10:** The comparison of MIP and GA.

Customers	Problem	Total cost		Elapsed time		Gap		Number of trucks		Number of TIRs	
		MIP	GA	MIP	GA	MIP	GA	MIP	GA	MIP	GA
10	1	9,400	9,900	340	42	—	5.32	4	2	2	5
	2	11,360	11,360	82	40	—	0.00	5	2	4	6
	3	10,700	11,120	131	40	—	3.93	3	2	5	6
	4	8,900	9,820	107	49	—	10.34	4	5	3	3
	5	11,820	12,400	100	45	—	4.91	4	3	5	6

15	1	18,140	18,640	7200	110	26.79	2.76	6	3	8	10
	2	14,100	14,940	7200	106	19.56	5.96	3	4	7	7
	3	13,600	14,900	7200	112	19.98	9.56	4	7	6	5
	4	16,100	17,520	7200	98	23.54	8.82	4	5	8	8
	5	15,020	15,980	7200	120	22.32	6.39	3	8	8	5

20	1	19,760	21,720	7200	217	6.37	9.92	1	3	12	12
	2	21,880	24,060	7200	209	4.77	9.96	0	7	13	11
	3	19,380	22,220	7200	216	6.47	14.65	2	8	11	9
	4	20,800	23,360	7200	204	6.88	12.31	2	5	12	12
	5	18,260	20,760	7200	202	5.75	13.69	1	5	11	10

30	1	29,740	36,120	7200	636	21.96	21.45	9	8	13	18

40	1	41,300	46,020	7200	1309	27.27	11.43	5	10	23	23

50	1	53,380	65,360	7200	3314	38.62	22.44	17	33	24	21

60	1	∗	79,020	7200	4371	100.00	∗∗	∗	21	∗	37

70	1	∗	81,920	7200	*7724 *	100.00	∗∗	∗	28	∗	34

80	1	∗	98,020	7200	*9932 *	100.00	∗∗	∗	30	∗	43

90	1	∗	118,440	7200	*15146 *	100.00	∗∗	∗	39	∗	50

			Avg.	5598	2011	37.08	9.66				

*MIP could not find any feasible solution within time limits.

∗∗Gap% values for GA could not be calculated because of the deficiency of MIP cells.

## Appendix

The pseudocode for the local search: for brevity, only the key steps are detailed. C# notation is used see Algorithm 1.
